# An organosynthetic soft robotic respiratory simulator

**DOI:** 10.1063/1.5140760

**Published:** 2020-06-09

**Authors:** Markus A. Horvath, Lucy Hu, Tanja Mueller, Jon Hochstein, Luca Rosalia, Kathryn A. Hibbert, Charles C. Hardin, Ellen T. Roche

**Affiliations:** 1 Institute for Medical Engineering and Science, Massachusetts Institute of Technology, Cambridge, Massachusetts 02139, USA; 2 Health Science and Technology Program, Harvard/Massachusetts Institute of Technology, Cambridge, Massachusetts 02139, USA; 3 Technische Universitat Munchen, D-80333 Munich, Germany; 4 Harvard Medical School, Boston, Massachusetts 02115, USA; 5 Massachusetts General Hospital, Harvard University, Boston, Massachusetts 02114, USA; 6 Department of Mechanical Engineering, Massachusetts Institute of Technology, Cambridge, Massachusetts 02139, USA

## Abstract

In this work, we describe a benchtop model that recreates the motion and function of the diaphragm using a combination of advanced robotic and organic tissue. First, we build a high-fidelity anthropomorphic model of the diaphragm using thermoplastic and elastomeric material based on clinical imaging data. We then attach pneumatic artificial muscles to this elastomeric diaphragm, pre-programmed to move in a clinically relevant manner when pressurized. By inserting this diaphragm as the divider between two chambers in a benchtop model—one representing the thorax and the other the abdomen—and subsequently activating the diaphragm, we can recreate the pressure changes that cause lungs to inflate and deflate during regular breathing. Insertion of organic lungs in the thoracic cavity demonstrates this inflation and deflation in response to the pressures generated by our robotic diaphragm. By tailoring the input pressures and timing, we can represent different breathing motions and disease states. We instrument the model with multiple sensors to measure pressures, volumes, and flows and display these data in real-time, allowing the user to vary inputs such as the breathing rate and compliance of various components, and so they can observe and measure the downstream effect of changing these parameters. In this way, the model elucidates fundamental physiological concepts and can demonstrate pathology and the interplay of components of the respiratory system. This model will serve as an innovative and effective pedagogical tool for educating students on respiratory physiology and pathology in a user-controlled, interactive manner. It will also serve as an anatomically and physiologically accurate testbed for devices or pleural sealants that reside in the thoracic cavity, representing a vast improvement over existing models and ultimately reducing the requirement for testing these technologies in animal models. Finally, it will act as an impactful visualization tool for educating and engaging the broader community.

## I. INTRODUCTION

Biohybrid robots are often designated as devices and machines that are actuated by living cells.^1^ Here, we reverse this paradigm and use artificial muscles to power passive biological tissue. In a recent review,^2^ the authors describe a robotic taxonomic key for biohybrid robots classified into whether organic components are used for structure, actuation, sensing, or control. Our proposal is to use soft robots where organic components are structural (lung tissue) and actuation is provided by synthetic components.

Motivated by the goal of reducing animal and human testing, the need for standardized high-fidelity, quantitative test methods for medical devices, and encouraged by the rapid advancements and accessibility in the field of soft robotics, we strive to develop realistic body-part simulators for the investigation of physiology, pathology, and interdependence of physiological systems and for the education and training of students and specialists with interactive high-fidelity simulation scenarios.

Due to the large number of variables surrounding respiration—including fluctuating volumes, pressures, flow rates, and compliances—mastery of respiratory biomechanics is a complex task. Key to the physiology of the respiratory system is the compliance of different elements. Compliance is defined as the change in the volume of a space due to a change in pressure, dV/dP. Our system allows for the tuning of compliance of different elements, such as the lungs or the abdominal cavity, via different soft material mechanisms. Respiratory pathologies often deal with compliance changes, so the tunability of compliance allows for an educational opportunity to examine the effects of isolated changes. Furthermore, intricate interventional strategies, such as mechanical ventilation, can be difficult for students to comprehend. However, as students progress into clinical training, robust biomechanical intuition is key for guiding accurate and decisive action from the clinical care team in the environment of critical care.

Medical simulators are key educational and training tools that can enhance understanding and intuition of complex biological systems, presenting a hands-on learning opportunity while causing no harm to a potential patient or a living animal. Simulating the mechanics of the respiratory system in a tunable model could, therefore, provide an active, experiential learning tool to aid in the development of an accurate workable mental model for clinicians in training.^3^ Although there exist a variety of simulators for medical training, none captures the extent of biomechanical and biophysical phenomena that govern the physiology of the respiratory system.

More specifically, medical simulators exist for a wide variety of applications, ranging from preclinical education to experiential training to medical technology testing and research.^3–7^ Perhaps the most commonly used educational respiratory simulator is the extremely simplified bell jar model of a balloon (representing the lungs) in a jar (representing the thoracic cavity) with an elastic membrane at the base of the jar (representing the diaphragm).^5,8^ When one manually pulls on the membrane, the negative pressure generated in the jar “inflates” the lungs. This is an inexpensive and accessible model; however, it fails to teach any of the more complex concepts involved in respiratory physiology. In clinical training, medical schools will often invest in complex mannequin-based simulators that rely on computational models of the relationship between clinical respiratory indicators.^3,4^ In using computational models to display the interactions of such indicators, these simulators compartmentalize the biomechanics into a “black box” model.^3,6^ These are effective in teaching clinical responsiveness and physical exam skills; however, they are extremely expensive (on the order of $150 000)^3^ and often a limited resource for institutions. In research and testing, respiratory simulators are largely created for the purpose of acting as dynamic radiological imaging phantoms.^9,10^ They focus on replicating dynamic motion of internal tissues (often focused on tumor motion with respiration for either the lungs or liver).^9–11^ In these systems, the motion is driven by servos and motors, with no utilization of pressure to drive air flow.

The most advanced respiratory simulator that replicates some mechanical principles of respiration in the pre-existing literature is now commercially available as the ArtiChest, which is marketed for use in endoscopic procedural training.^12^ ArtiChest does drive respiration via inducing negative pressure in the thoracic cavity, utilizes fresh animal lungs (porcine or ovine), and drives a “diaphragm” balloon via pressurizing the “abdominal cavity.” However, by utilizing a passive diaphragm in which upward motion is driven by increases in abdominal pressure, it inverts the normal relationship between abdominal and thoracic pressures. In patients, increasing abdominal pressures can impede respiration. ArtiChest does not track any pressure waveforms or display the effects of tuning the compliance of different elements in the system. Ultimately, ArtiChest's focus as a procedural simulator limits its utility as a preclinical educational tool.

Each of these existing simulators has a narrow application focus. None has been developed to fully explore the mechanics that drive respiration. For the most part, they focus exclusively on the thoracic cavity, neglecting the interdependence of the thoracic and abdominal cavities, which are coupled by the active but flexible muscle of the diaphragm. Considering that the diaphragm normally drives up to 70% of inspiratory efforts,^13^ this simplification ignores key interactions that help explain the biomechanics of respiration. Few existing *in silico* models of diaphragmatic motion and resultant pressures are computationally intensive and insufficient for rapid prototyping. Currently, no *in vitro* models exist that can replicate breathing pressures based on the movement of the diaphragm alone. Therefore, we aim to develop a simulator that is not application based but instead focused on replicating the biomechanics of this system. As such, this simulator has utility in medical education as well as in research and testing. This system is currently configured to serve as a versatile platform technology that can be adapted for intended use. Due to the modular nature of the system, specific elements can be adjusted to focus on the exact needs of different applications, such as creating an active diaphragm insert to study the diaphragm dysfunction or the integration of cardiovascular flow loops to create a cardiopulmonary simulator. Currently, there are no existing simulators that are set up to be able to capture the intricate interplay of all these systems. Robust *in vitro* simulators can enable rapid prototyping in the medical device design process without the use of living creatures.

**FIG. 1.**
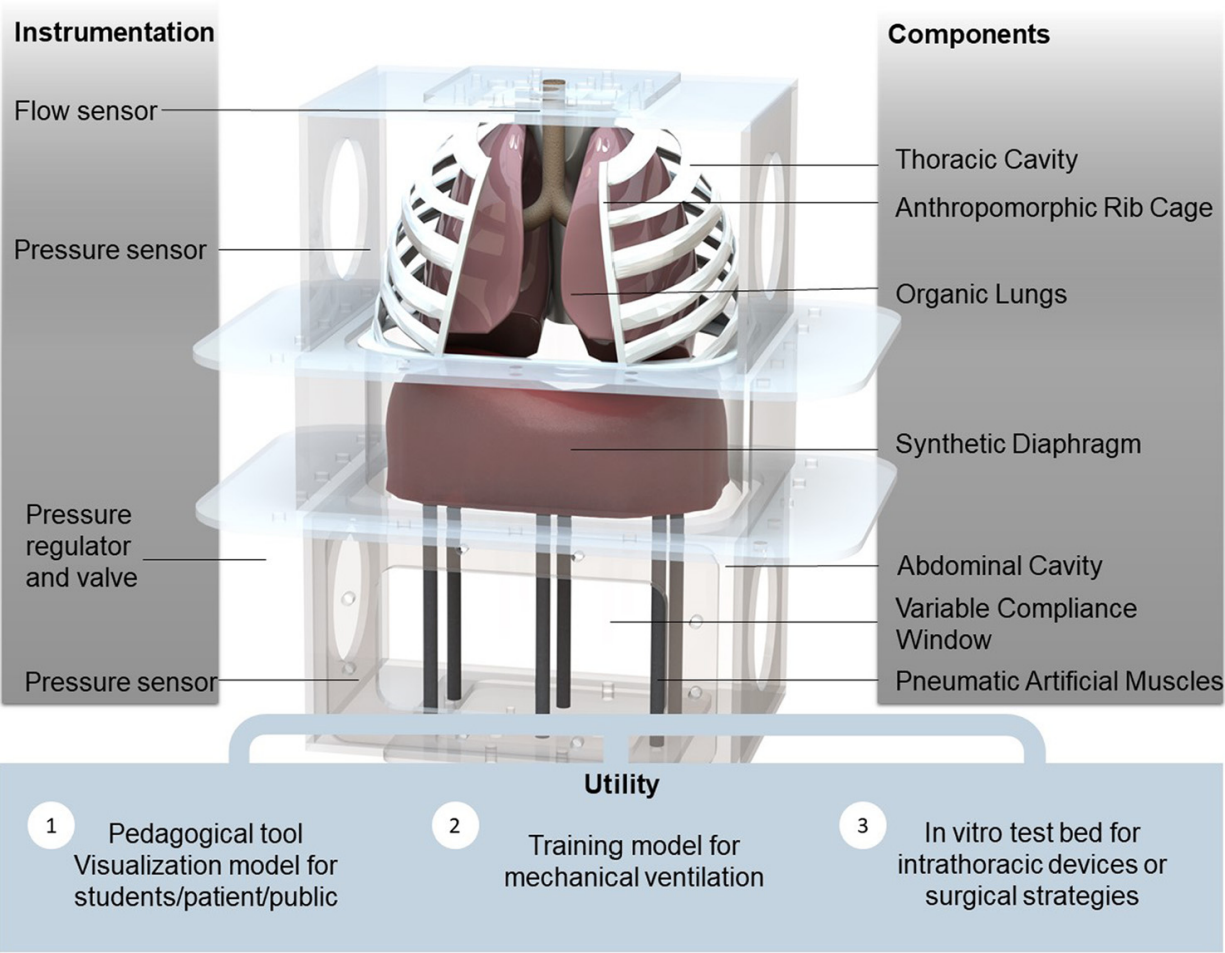
A computational rendering of our biohybrid simulator focused on replicating respiratory mechanics. By tracking pressures and flows via included pressure sensors, the simulator may be utilized for a variety of education and training purposes.

As soft robotics are effective in replicating controlled muscle motion, we sought to introduce mechanically programmable soft robotics into a dynamically pressurized system replicating diaphragm displacement, and by doing so, we ventilate lungs in the thoracic cavity. The modular nature of the system we developed allows customization of different features that are important for various application specific uses. For example, in cases in which replicating parenchymal tissue properties of the lungs is critical, organic lungs can be integrated, thus combining the advantage of biologically accurate tissue mechanical properties with the controllability of the robotic system. In addition, we are able to track the pressures in both the thoracic and abdominal cavities, and the use of various silicone elastomers and soft robotic elements—i.e., the pneumatic artificial muscles (PAMs) that are used to pull the diaphragm, seen in Fig. 1—allows us to tune the compliance of multiple components of the system in order to replicate physiologic pressures and volumes and therefore actively replicate the expected biomechanics of respiration. Soft robotic approaches include materials with moduli comparable with those of soft biological materials. Moduli of silicone and rubbers traditionally range between 10^4^ and 10^8^, which corresponds to the range of moduli for biologic materials from fat (∼10^4^ Pa) to cartilage (∼10^6^ Pa) and skin (∼10^7^–10^8^ Pa).^14^ Silicone materials have been used previously to tune the compliance of mock blood vessels in circulatory simulators of the pulmonary system.^15^ To the best of our knowledge, there is no previously reported work utilizing tunable elastomeric materials to modulate compliance elements in the context of respiratory simulators.

## II. RESPIRATORY MECHANICS

This simulator focuses on replicating physiological pressures and volumes by recreating the mechanical interactions of the lungs, diaphragm, pleural space, and abdomen.

**FIG. 2.**
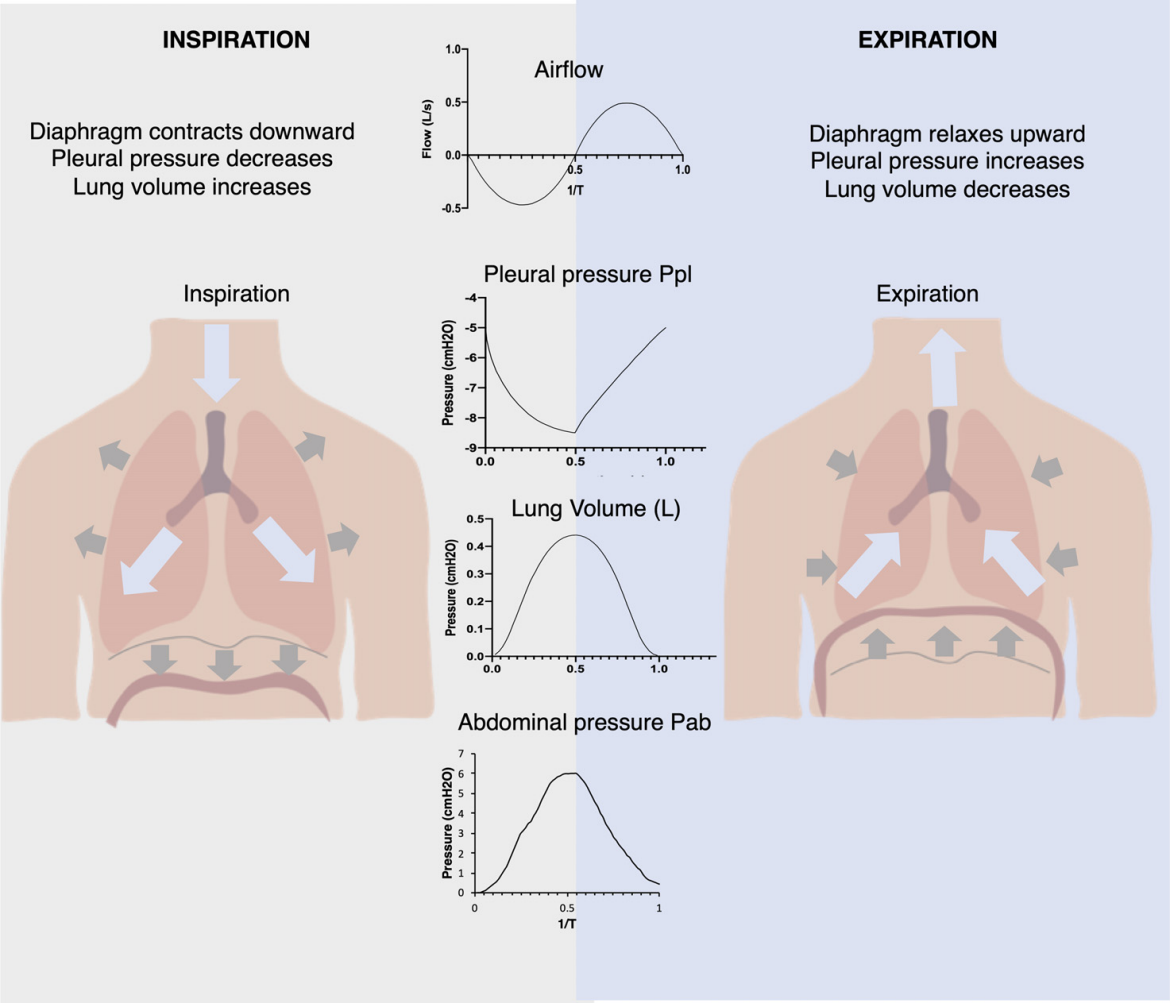
Schematic of how diaphragm displacement affects the pressures in the respiratory system driving airflow generating changes in the volume of the lungs, providing for the gas exchange of inspiration and expiration.

The diaphragm is the major muscle responsible for inspiration, contributing the majority of pump function within the respiratory system.^16^ In normal tidal breathing, downward motion of the diaphragm increases the volume of the thoracic cavity, decreasing pleural pressure (Ppl)—baseline –3 to –5 cm H_2_O, with quiet breathing efforts generating pleural pressures down to –9 cm H_2_O^17^—and alveolar pressure (Palv) to below the pressure at the airway opening and, thus, driving airflow into the lungs. The diaphragm also couples the thoracic and abdominal cavities together, as its downward motion simultaneously decreases the volume of the abdominal cavity with increasing abdominal pressure (Pab). Clinically, the most relevant pressure outputs are the pleural pressure and the abdominal pressures (see Fig. 2). The pleural cavity is the potential space between the tissue lining the exterior surface of the lung and the interior surface of the thoracic cavity. In our system, this corresponds to intrathoracic pressure. We aim to match the physiologic pressure waveforms for the pleural and abdominal pressures to clinical data for normal breathing.

## III. RESULTS AND DISCUSSION

### A. Replication of breathing pressures is enabled by our simulator model

We realize this simulator as an *in vitro* benchtop setup equipped with pressure and flow sensors and tunable mechanical properties. We constructed the chassis of the simulator out of optically clear plastics that act as the torso (Fig. 1). The chassis encloses the thoracic and abdominal cavities, separated by a diaphragm. This simulator is designed to be modular, and therefore, we included rapid-access ports on the sides of the acrylic boxes. The top and bottom sides of the chassis include modular tubing ports to allow for pressure controls and measurements. Our simulator assumes a rigid rib cage. The rib cage is broken into two zones: the upper 3D-printed ribs, which act as a boundary between the lungs from the outer thoracic cavity space, and the lower plastic shell, which aims to mimic the zone of apposition (a vertical area of the diaphragm that begins at the insertion point on the inside of the lower ribs and extends to the top of the domes). Because the abdominal cavity is a much more compliant enclosure compared to the rib cage, the abdominal cavity contains a modular compliance window—a critical feature that allows us to tune the relationship between the thoracic and abdominal cavity pressure waveforms.

**FIG. 3.**
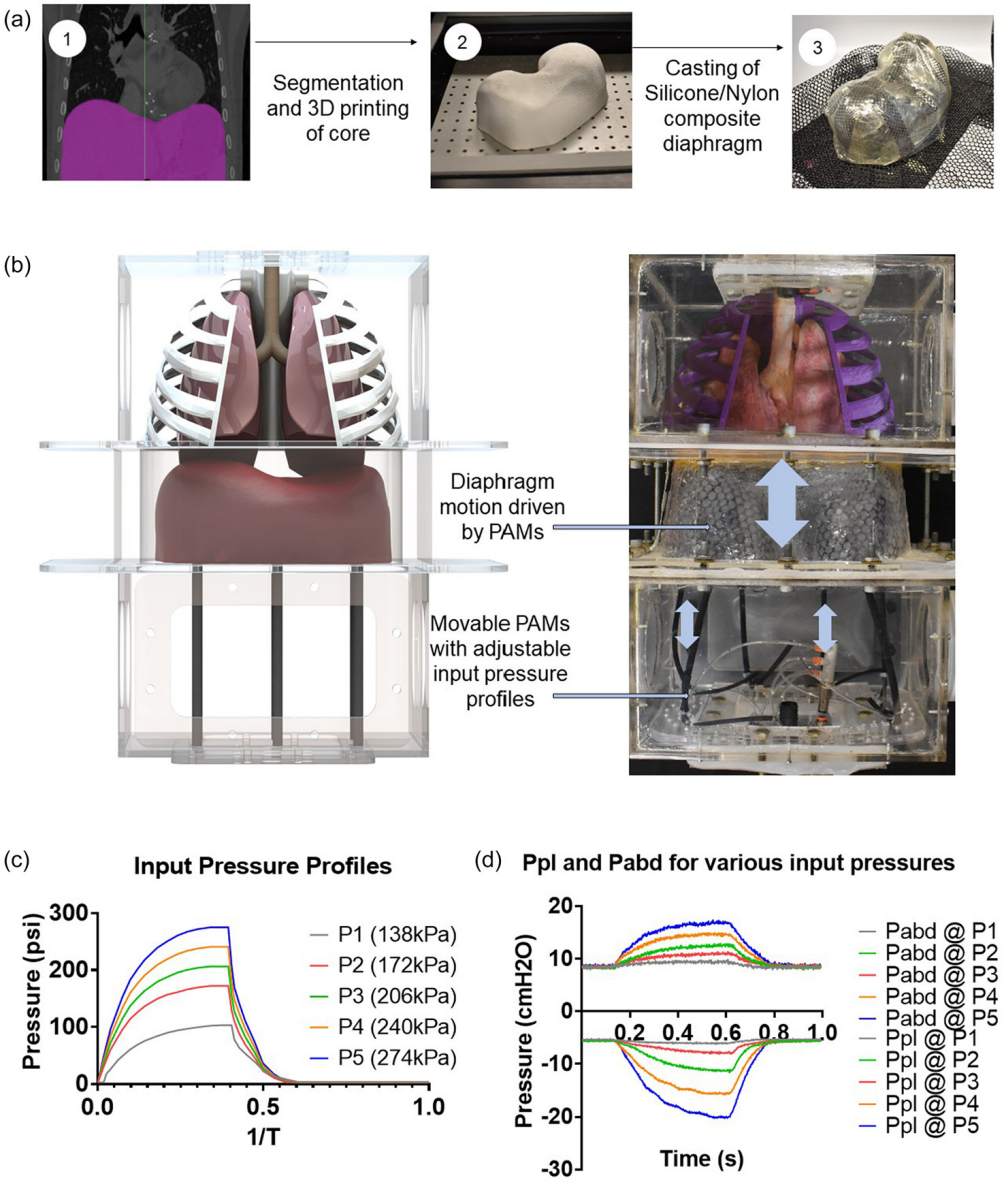
(a) Physical diaphragm insert from 3D data derived from clinical imaging using Mimics software. (b) Rendering and physical realization of the biohybrid respiratory simulator. (c) Pneumatic artificial muscles with different input pressures generate different degrees of diaphragm displacement. (D) Measured outputs of abdominal and pleural pressure in response to varying actuator pressures.

To generate respiration, we drive the motion of the diaphragm. The native diaphragm is a flexible but active muscle that generates up to 70% of the inspiratory tidal respiration^13^ via volume changes of the thoracic cavity. As a membrane, it couples the pressures of the thoracic and abdominal cavities together. In our simulator, we represent diaphragm motion via a flexible, passive silicone membrane that is moved via active pulling soft robotic elements located in the abdominal cavity. See Sec. V for further details. The diaphragm displacement is driven by pneumatic artificial muscles (PAMs) pulling on the silicone diaphragm. The PAMs are actuated via a custom electro-pneumatic control box that allows us to program specific actuation schemes and is described in the supplementary material. By varying the actuation schemes and, thus, the actuator contraction, we can mimic shallow and deep breaths. We can achieve a range of physiological breathing pressures, generating pleural pressure between –20 and –6 cm H_2_O, seen in Fig. 3. To characterize the pleural and abdominal pressure ranges of the simulator, no rib cages or lungs were included initially. As shown in Fig. 3(d), the contraction of the diaphragm drives the pleural pressure to be more negative and the abdominal pressure to be more positive. This difference is referred to as the transdiaphragmatic pressure and is a metric for diaphragm effort and function.

### B. Addition of organic lungs to realize a biohybrid respiratory simulator

**FIG. 4.**
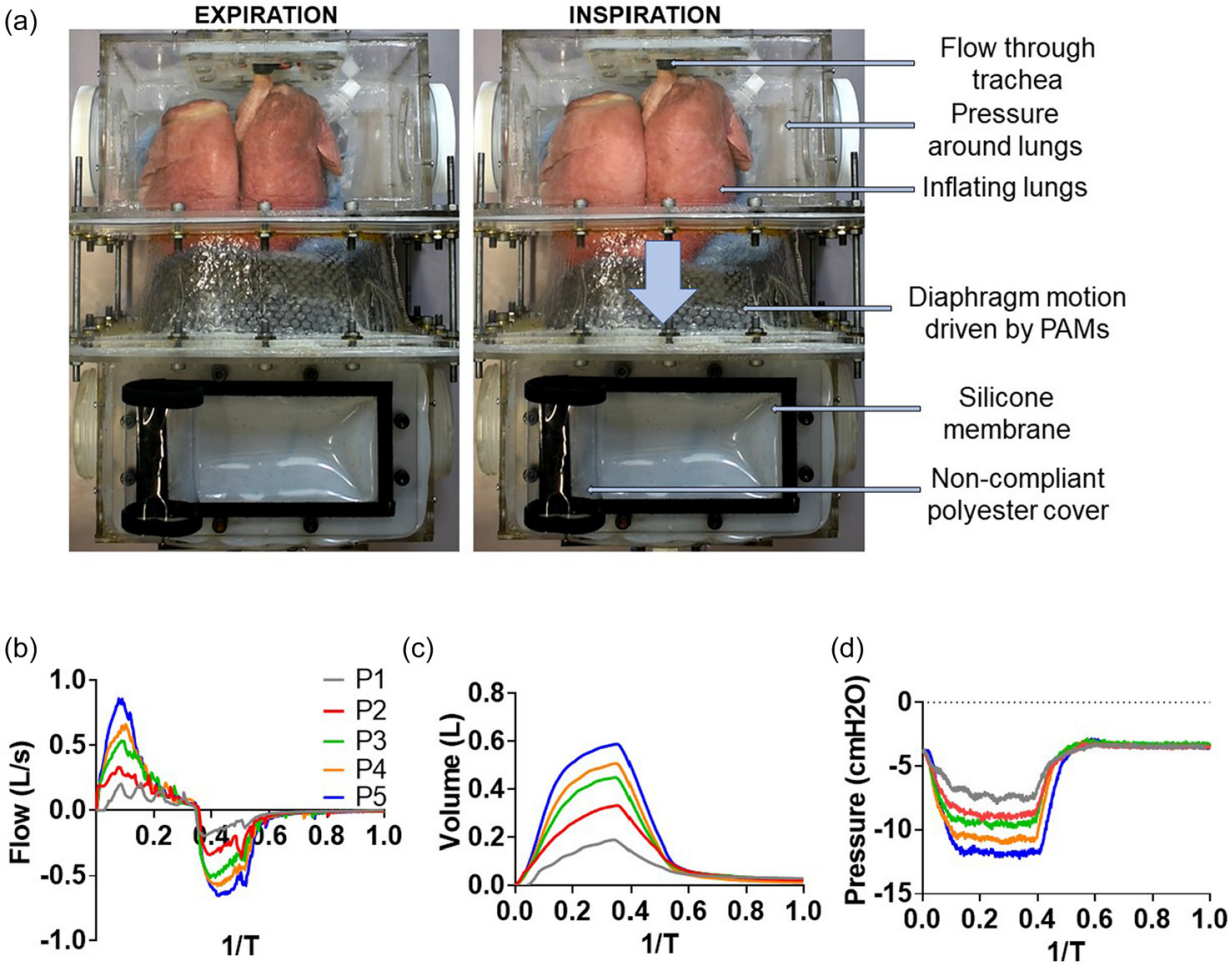
Inclusion of organic lungs to visualize respiration and measure airflow. To visualize lung motion, the rib cage was removed. (a) When the artificial muscles are not contracted, the diaphragm is in its resting state. With downward displacement of the diaphragm, inspiration and lung expansion are observed. Spirometry readings replicate physiologic waveforms for flow (b), volume (c), and pleural pressure (d). Multimedia view: http://dx.doi.org/10.1063/1.5140760.1
10.1063/1.5140760.1

In order to replicate the mechanical properties of lung parenchyma, this simulator can be coupled with organic lungs, enabling the measurement of respiratory flows and volumes. Varying the degree of diaphragm effort as described above allows us to generate a range of physiological flows, tidal volumes, and pleural pressure as shown in Fig. 1. Our flow waveforms are rather tortuous; these spiking waveforms are an artifact of the highly sensitive internal control system of the electropneumatic regulators that our system utilizes. Due to the signal-to-noise ratio of the pressure control at low pressure actuation—evidenced by the gray line in Figs. 4(b)–4(d)—the oscillatory effects of the control system are more pronounced. However, because the more relevant clinical metric is volume, the time integral is smooth enough to closely match the physiologic waveforms. Additionally, organic lungs allow for the visualization of inflation and deflation, seen in Fig. 4 (Multimedia view). As an educational tool, this simulator gives students a visual intuition for these internal systems.

### C. Varying compliance of each component of the simulator

The pressures of the respiratory system interact through various organ and tissue systems, each with unique mechanical properties. The ability to vary compliance of different components in this respiratory simulator allows us to tune the performance of the simulator to match both physiologically normal conditions and also to examine the mechanical effect that pathologic changes to these compliances have on respiration.

**FIG. 5.**
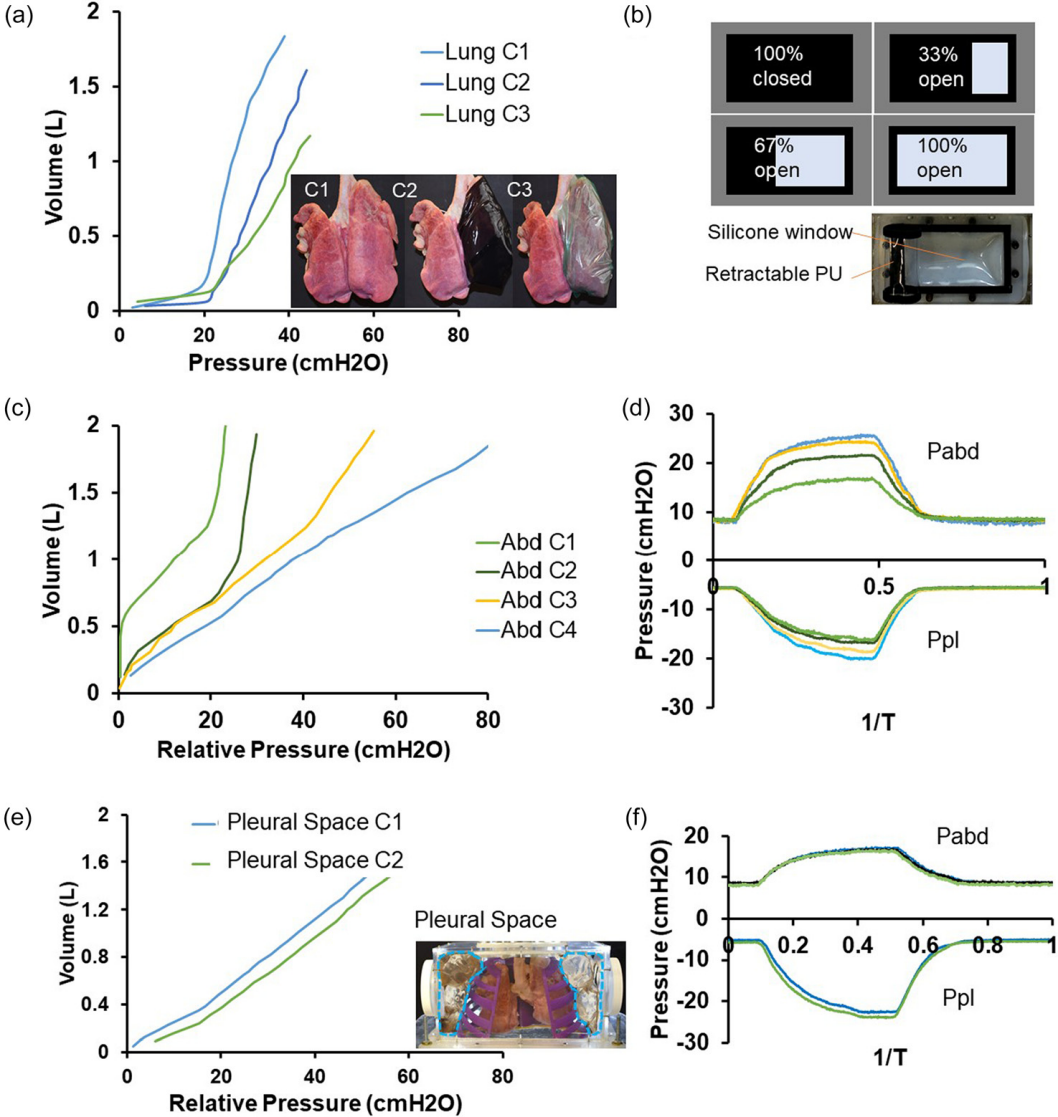
Modifying the compliance of different elements. (a) Varying lung compliance. (b) Schematic of tunable compliance of the abdominal cavity via the silicone window. (c) Varying abdominal cavity compliance. (d) Effect of variable abdominal cavity compliance on abdominal and pleural pressures during 1 cycle of respiration. (e) Varying pleural cavity compliance. (f) Effect of variable pleural cavity compliance on abdominal and pleural pressures during 1 cycle of respiration.

Specifically, we can individually vary the effective compliance of the lungs, pleural space, and abdominal cavity and subsequently measure the effects of respiratory volumes and pressures, seen in Fig. 5. We use organic porcine lungs to represent the compliance of “normal lung tissue.” This compliance can be decreased by wrapping the lungs in externally restrictive materials: C2 is created with a semi-distensible film around the lungs and C3 is created with a non-distensible film around the lungs [see Fig. 5(a)]. The decreased compliance mimics a net stiffening of the lungs in a restrictive lung disease.

The compliance of the abdominal cavity ultimately affects the relationship between the pleural and abdominal pressures, and thus, a silicone window with a variable non-distensible covering allows the creation of various compliances [see Figs. 5(b)–5(d)]. The variable compliance window of the abdominal cavity consists of a highly compliant silicone sheet that can be partially blocked by a stiff, polyurethane window covering to decrease the compliance: C1 is created with the window completely open, C2 is created with the window 33% open, C3 is created with the window 67% open, and C4 is created with the window completely closed. This can be finely tuned to mimic the effect of higher abdominal muscle tone. We can thereby control intra-abdominal pressure and mimic pathophysiological conditions like abdominal compartment syndrome. Additionally, selective actuation of the pneumatic elements contracting the diaphragm model downward can serve as a model for unilateral diaphragm paralysis.

Although we assume a rigid ribcage, we represent changes in pleural cavity compliance by modulating the volume of gas between the lungs and chest walls. Because there are no defined pleural cavities and the pressure is equal throughout the space, we modulate the space external to the 3D-printed rib cage even though it is different from the native anatomy. By filling the space with an incompressible fluid, we replace the compressible air from the pleural space: C1 is created as the air fills the volume around the lungs and C2 is created with partially filled sacs surrounding the lungs [see Figs. 5(e) and 5(f)]. This decreases the overall compliance of the volume surrounding the lungs.

The ability to independently control the compliance of different elements enables selective adjustment of compartment pressures in the thorax and abdomen and isolation of different mechanical phenomena for educational purposes. By examining the effects of each of these variables independently and then combining together, this simulator allows students to generate robust biomechanical mental models of the respiratory system.

### D. Replication of pathologic conditions

**FIG. 6.**
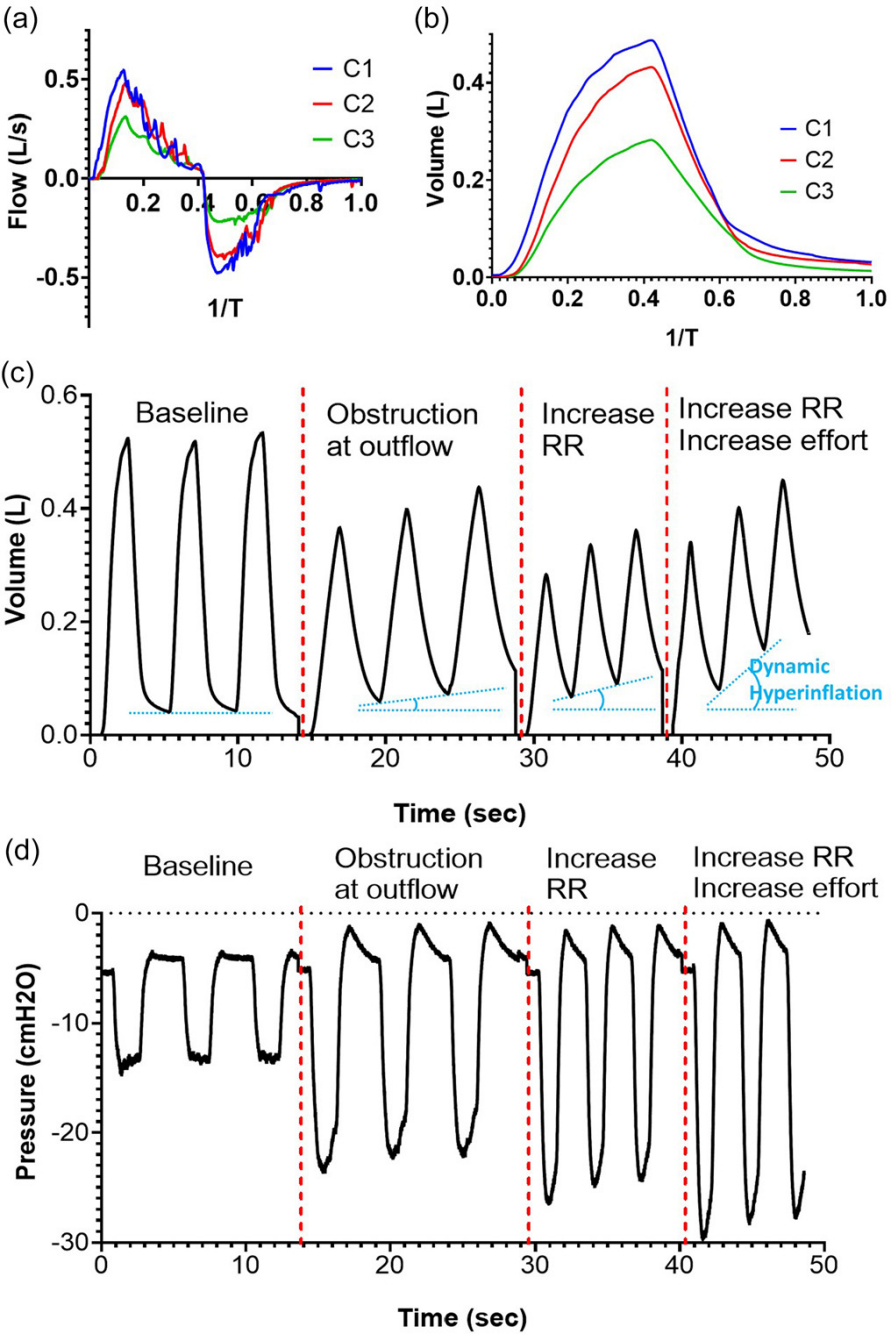
Simulating restrictive and obstructive lung disease. (a) Decreased flow to the lungs during a breathing cycle in modeling restrictive lung disease. (b) Reduced tidal volumes during a breathing cycle in modeling restrictive lung disease. (c) Lung volume waveforms in simulating dynamic hyperinflation via increasing resistance, respiratory rate, and diaphragm effort. (d) Pleural pressure waveforms in simulating dynamic hyperinflation via increasing resistance, respiratory rate, and diaphragm effort.

The compliant elements of the simulator are first tuned to generate physiologically normal biomechanics. Once the typical values are established, the modular nature of the simulator allows for the appropriate variables to be adjusted to replicate pathologic conditions. A key concept in preclinical respiratory physiology is that of restrictive and obstructive lung disease. Intrinsic restrictive lung disease, such as pulmonary fibrosis, often occurs due to stiffened compliance changes to the lungs that prevent the necessary expansion. We demonstrate the ability to create stiffened lung tissue in Figs. 6(a) and 6(b); our simulator can output decreased pressures and flows resulting from the compliance change we induced to the lung tissue as shown in Fig. 5(a). In Figs. 6(a) and 6(b), we examine the flow and volume waveforms of the simulator, and we show that given the same diaphragm effort, increased lung stiffness decreases the ventilation capacities of the system. This can give insight into the pathophysiology of restrictive lung disease.

Obstructive lung disease is due to an increase in flow resistance in the respiratory system, such as the narrowing of the bronchioles in asthma or chronic obstructive pulmonary disease (COPD). We can mimic the effect of increased flow resistance by adding a flow resistor in series with the airway, seen in Fig. 6. Increased resistance of the system leads to an increase in the emptying time of the lungs. If the respiratory rate increases such that there is an insufficient amount of emptying time between breaths, subsequent breaths introduce an additional volume and additive pressure leading to dynamic hyperinflation. Figure 6(c) shows the decrease in the tidal volume with the introduction of an obstruction, the subsequent decrease in the tidal volume, introduction of dynamic hyperinflation with the addition of an increase in the respiratory rate, and the worsened dynamic hyperinflation with the addition of an increase in diaphragm effort. Figure 6(d) shows the decay curve during expiration that occurs due to the added resistance, effectively increasing the RC time constant of the system. The increase in the respiratory rate introduces dynamic hyperinflation, which results in the buildup of pressure, and increases the minimum possible pressure generation, which ultimately requires increased diaphragm effort in order to generate the extremely negative pressures to compensate for the low tidal volume. These graphs elucidate the complexities of the respiratory mechanics that occur during obstructive lung disease.

**FIG. 7.**
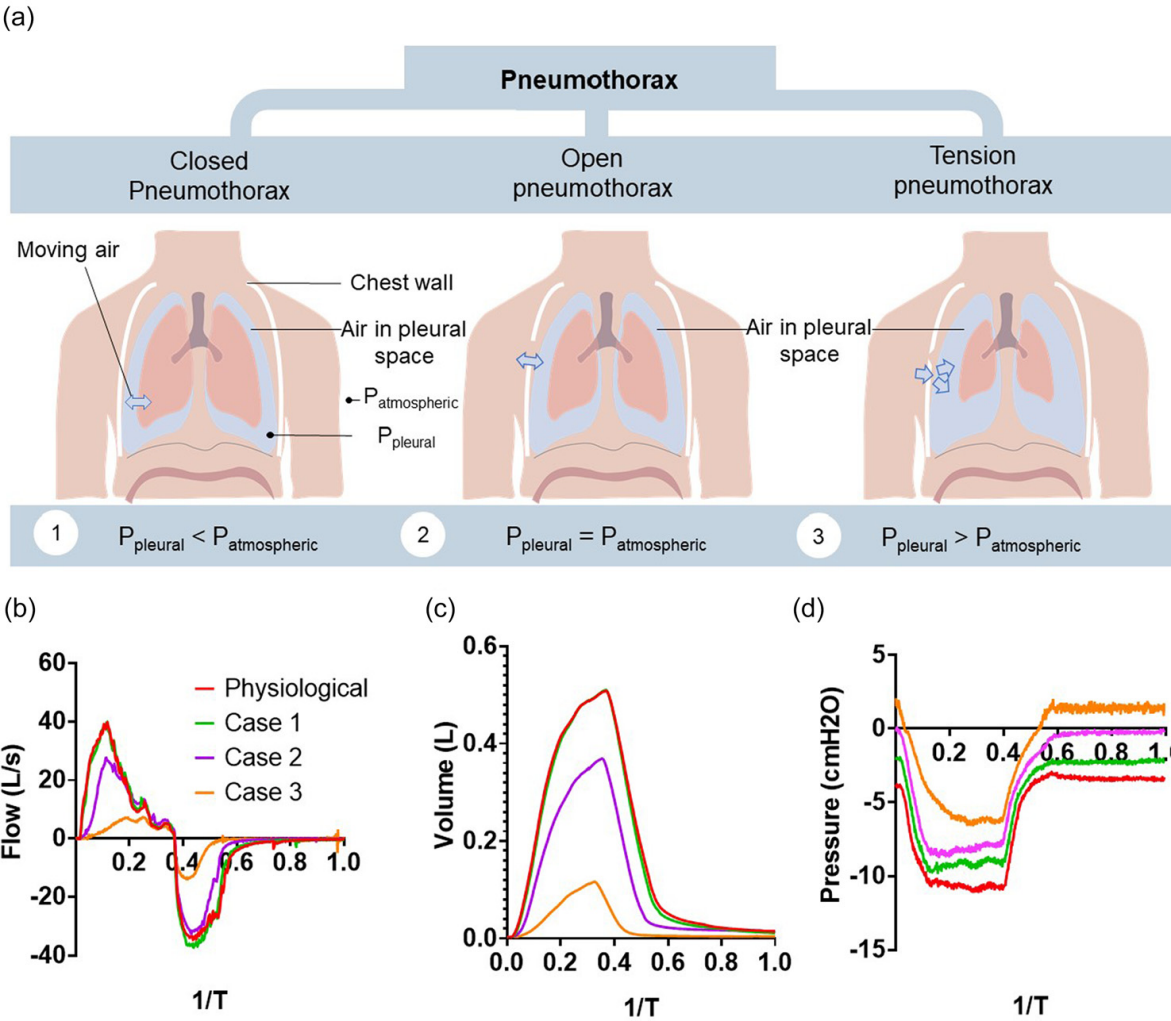
Simulating the mechanical effects of a pneumothorax. (a) Schematic depicting the different categories of pneumothorax. (b) The ventilatory airflow measured for varying degrees of a pneumothorax. (c) The tidal volume generated for the varying degrees of a pneumothorax. (d) The measured pleural pressure in the varying degrees of a pneumothorax.

Another pathology we can replicate is the case of a pneumothorax, when air improperly enters the pleural space, disrupting the pleural pressure, seen in Fig. 7(a). There are broadly three types of pneumothorax—closed, open, and tension pneumothorax—which are determined by the degree of disturbance to pleural pressure. A closed pneumothorax occurs when air inappropriately enters the pleural space, but the pleural pressure remains negative. When the pleural space has equalized in pressure to the atmosphere, this is described as an open pneumothorax. A tension pneumothorax occurs in which a valve like opening in the chest wall leads to trapping of air in the pleural space to the point where the pleural pressure is positive. This disruption can lead to a collapsed lung, which requires a chest tube to restore the negative pressure of the pleural space. To simulate the varying degrees of a pneumothorax, we set the baseline pleural pressure to different levels while keeping diaphragmatic efforts constant. Figures 7(b)–7(d) show the nonlinear sensitivity of the respiratory system to the degrees of a pneumothorax. Cases 1 and 2 reveal minimal changes in the respiratory mechanics, displaying the general robustness of the respiratory system in which minor changes in initial pleural pressure do not affect the ventilation. We find that case 3 and beyond start to generate noticeable effects, delineating the inflection point at which a pneumothorax begins to have severe effects on function, demonstrating the difference in severities of the different categories of a pneumothorax. We find that in case 4, those simulating a tension pneumothorax, we see extremely diminished lung filling capacity, as the lung struggles to expand against the pressurized pleural space. At a baseline pleural pressure of +5.5 cm H_2_O, we find that the diaphragm displacement is unable to generate airflow or changes in the lung volume and pleural pressure without increased breathing efforts.

### E. Utility of the simulator for critical care training

**FIG. 8.**
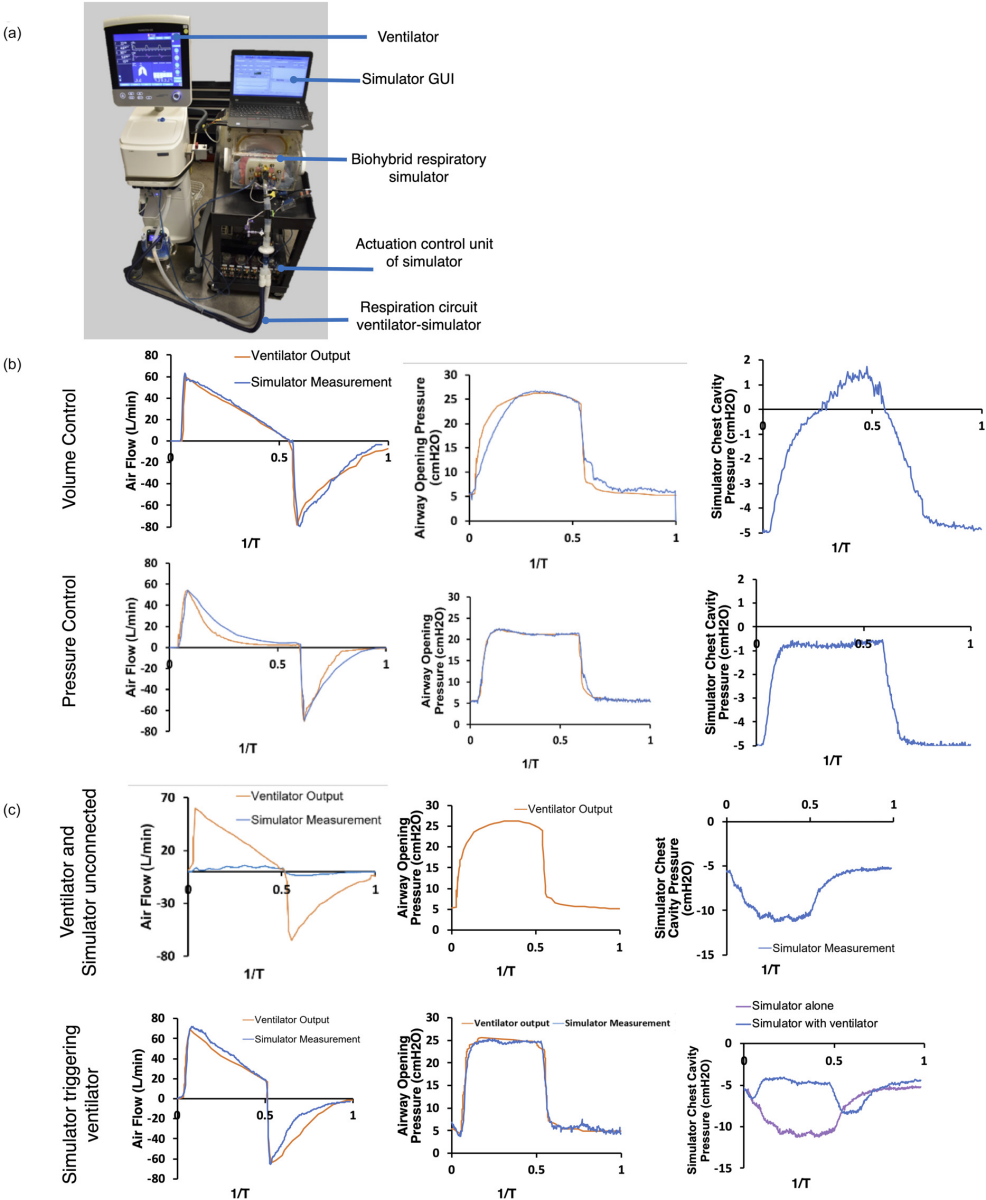
Integration of the respiratory simulator with a mechanical ventilator. (a) Image of our experimental setup combining the simulator with an existing mechanical ventilator. (b) Flow and pressure measurements in which respiration is driven only by the mechanical ventilator. (c) Flow and pressure measurements when the ventilator is disconnected, and the simulator is driving a low tidal volume breath. (d) Flow and pressure measurements when the simulator drives a low tidal volume breath that triggers a breath on the coupled ventilator.

A core component of training for both medical students and resident physicians in critical care work is understanding how to operate and set positive pressure mechanical ventilation. Mechanical ventilation is a key life-saving technology, but its many settings require proper understanding in order to provide adequate therapy. Improper ventilator settings can cause discomfort or even additional harm, such as pulmonary barotrauma. Our respiratory simulator can integrate with any existing mechanical ventilator to provide a risk-free and tailored learning environment. The goal of the simulator is not exact recapitulation of idealized waveforms, but instead demonstrating that the simulator can integrate well with existing clinical hardware for training purposes, seen in Fig. 8.

By operating the simulator without the active diaphragm displacement, we can demonstrate the effect of ventilator-only respiration, seen in Fig. 8(b). Driving respiration only with the mechanical ventilator, we validate our flow sensor outputs and compare them with the ventilator's sensor outputs for both volume-controlled and pressure-controlled ventilation. Our respiratory simulator gives additional insight into the effect of positive pressure ventilation on the pleural cavity pressure. This can provide clarity as to the distinctions in between different ventilator-modes, such as volume-controlled vs pressure-controlled ventilation. The simulator can also be operated with synchronized controlled mandatory ventilation as seen in Figs. 8(c) and 8(d). This is an example for ventilator modes that sense inspiratory efforts by the patient and support them by delivering a volume controlled tidal volume. The respiratory simulator can be operated independent of the ventilator with a low level of breath, which would not suffice for a full physiologic tidal volume. The different driving mechanisms of ventilation can be seen in the baseline waveforms of the ventilator and simulator operating independently and uncoupled, which are shown in Fig. 8(c). When connected, the same low volume breath can trigger the ventilator in the synchronized controlled mandatory ventilation mode. Inspiratory efforts of the simulated patient trigger the delivery of a pre-defined tidal volume by the ventilator, creating an additive effect of the simulator effort and ventilator effort seen in the pressure of the pleural cavity in Fig. 8(d). The respiratory simulator is able to be synchronized with the ventilator and trigger breath delivery as a patient would. Additionally, our simulator reveals a measurement that is not gathered during regular operation of a mechanical ventilator—the pleural pressure. The value of measuring the pleural pressure, which is possible in our simulator, but difficult clinically, is in the ability to deduce the transpulmonary pressure (i.e., the difference between airway pressure and pleural pressure) rather than assuming that the transpulmonary pressure is well approximated by the airway opening pressure. The increased intrathoracic pressure can induce cardiovascular insufficiency in patients with hypovolemia due to the decreased venous return.^18^ The decrease in cardiac output is often a dramatic sudden decrease in function but can normally be reversed by fluid resuscitation. Notably, the airway waveform is less relevant to the hemodynamics than the effect of intrathoracic pressure. Therefore, our simulator provides the more relevant mechanical variable that is not directly measurable in the clinical setting. This provides an opportunity to offer a clarifying explanation to a challenging concept. The modular nature of the system allows us to adjust what outputs are visible, and so after the concept is clarified, the intrathoracic pressure reading can be removed to simulate operation with only the variables available in a clinical context. With this visualization, the simulator can expand a trainee's mental model of the system. This model enables rapid understanding of the mechanics that allows clinicians to make smooth and decisive interventions, such as fluid resuscitation, prior to crisis.^18^ Our simulator, therefore, offers a key window into this complex biomechanical system by enabling the accurate measurement of pressures that are not routinely measured clinically.

### F. Utility of the simulator for interventional research

**FIG. 9.**
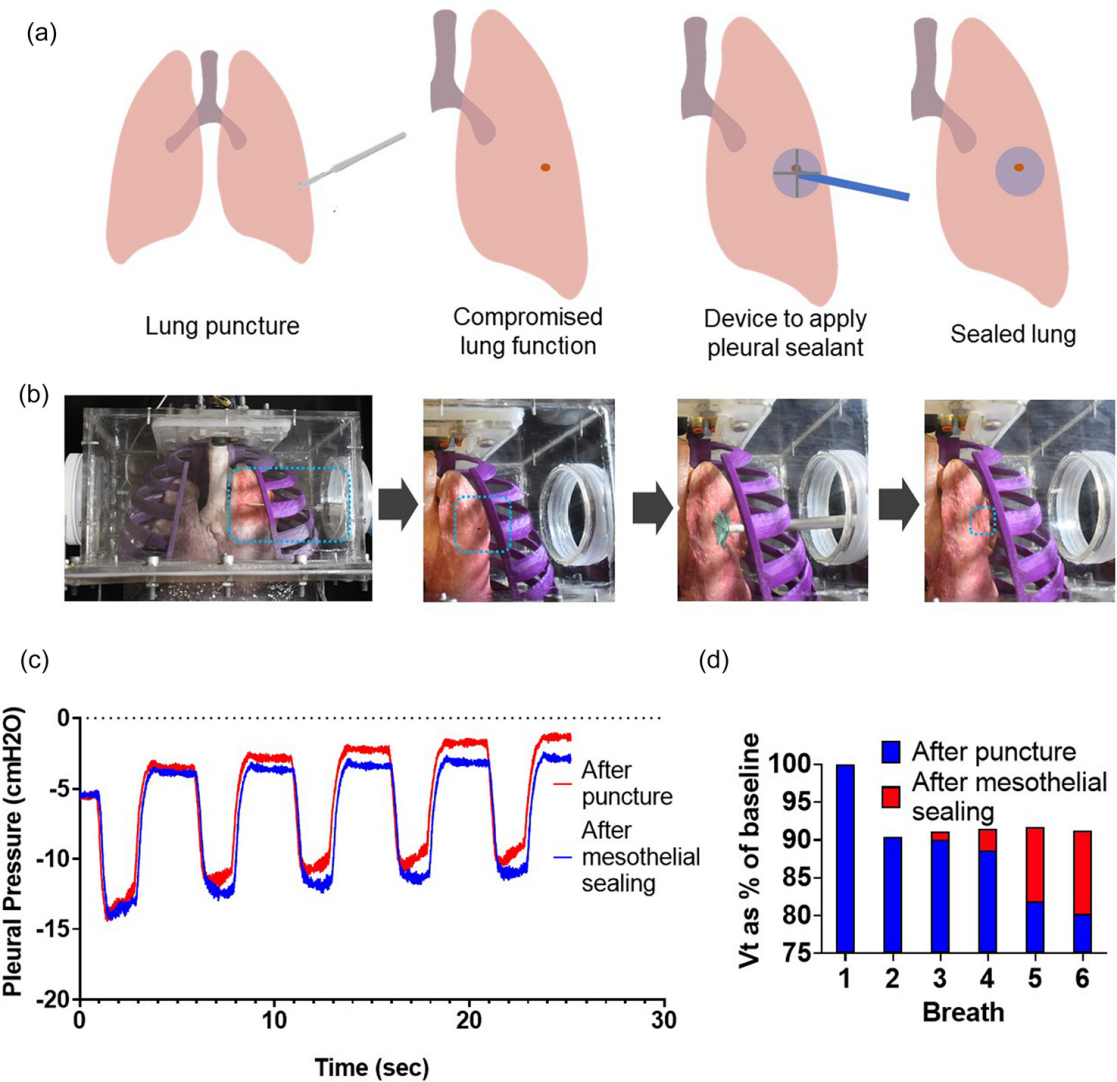
Use of the respiratory simulator for medical device testing. (a) Schematic of the procedure to test the ability of a mesothelial patch to seal a lung puncture. (b) Images of the experimental procedure. (c) Pleural pressure over multiple breaths and (d) the tidal volume over multiple breaths.

The necessity of a negative pressure in the thoracic cavity often poses a challenge for interventional medical device research. Animal cadaver studies are often an ideal prototype testing arena for medical device development, providing an inexpensive opportunity to refine the technology prior to conducting *in vivo* animal trials and reducing the total amount of animal testing. Unfortunately, in the case of interventional respiratory technologies, it is difficult to surgically access the thoracic cavity and subsequently restore the negative intrathoracic pressure in an animal cadaver. Our simulator provides a benchtop testing platform that can replicate respiratory mechanics, enabling better *in vitro* and *ex vivo* testing before moving to *in vivo* models. One such technology that benefits from our platform is the development of a sealing patch for lung puncture repair during thoracic surgery.^19–21^ The current performance metric for this technology is pressure-decay testing, which is the measured airway opening pressure as the lungs are pressurized via orotracheal intubation to a set level, and then the ability to maintain that pressure is measured. In our simulator, we test the ability of a mesothelial patch to seal a puncture in the organic porcine lungs seen in Fig. 9. Figures 9(a) and 9(b) show the process of testing the sealant; further details can be found in Sec. V. In addition to measuring airway opening pressure, Fig. 9(c) shows that we have the ability to monitor the pleural pressure. We see the increasing pleural pressure from the leak that is a result of the punctured lung and show the ability of the patch to minimize the leak. Over the course of five breaths, the punctured lung leads to a rise in pleural pressure to –1.5 cm H_2_O, while the mesothelial sealing manages to maintain a negative pleural pressure of –4 cm H_2_O. In Fig. 9(d), we find that after puncture, there is rapid loss in the tidal volume generated in subsequent breaths, and we show that the patch has the ability to preserve 90% of tidal volume, whereas without sealing, the tidal volume decays to 80% of the baseline, more clinically relevant metric than pressure-decay testing. This demonstrates how the sensors in our simulator provide a robust and quantitative metric for evaluating the success of the patch.

## IV. CONCLUSIONS

We demonstrate a respiratory simulator that replicates the biomechanics of ventilation that functions as an educational, training, and research tool. Our simulator drives diaphragm displacement with soft robotic actuators. By varying the contraction of these actuators, we can vary diaphragm effort to generate a spectrum of physiological pressure waveforms for the pleural and abdominal cavities. By integrating organic lungs, we can replicate ventilatory flow and tidal volumes in this bio hybrid simulator. We can independently vary the compliance of different components of the respiratory system to be finely tuned to match the physiological mechanics.

As an educational tool, this simulator replicates the mechanical physiology of the system—showing the interdependence of pressure, volume, compliance, and flow—and grounds clinical concepts in biomechanics, providing a robust mental model for students. The modular nature of our simulator allows for not only the replication of normal physiologic motion but also simulation of a host of pathologies, sourced back to their mechanical dysfunction. The compliances can be further adjusted to simulate pathologies where these are altered, as in including restrictive lung diseases. We can adjust the resistance of the airway tree and model the effects of obstructive lung disease, such as dynamic hyperinflation. By modulating the baseline pressure in the pleural cavity, we can model the effects of different degrees of a pneumothorax on respiration. As such, this simulator has utility not only in preclinical education but also in more advanced clinical training. For training of more advanced concepts, we can integrate the simulator with existing mechanical ventilators. When coupled with a mechanical ventilator, this simulator also acts as a platform to investigate the effect of different ventilator settings, providing a physical intuition between the different modes and functions and the mechanics of the respiratory system. The simulator can integrate seamlessly with different modes of mechanical ventilation including patient initiated assisted ventilation

Additionally, we demonstrate the research utility of this simulator as a testbed for new device technology via a mesothelial sealing patch. The simulator enables the testing of devices that interact directly with the negative intrathoracic pressure and filling of the lungs, which are difficult to examine in animal cadaver studies. The simulator also enables the collection of metrics to evaluate the technology that are both more quantitative and more sensitive than the existing metrics.

Our simulator has limitations due to some of the simplifying assumptions made. We can couple the simulator with either silicone or *ex vivo* lungs, depending on the importance of replicating parenchymal properties. If the focus of a simulator exercise is examining diaphragm mechanics, silicone lungs can be used as a simplification of the system for ease of use. In the case of using *ex vivo,* freshly sourced, organic lung tissue to replicate the parenchymal properties, the simulator is subject to the natural inter-organism variabilities of the lung tissue. Additionally, these lungs lack pleural membranes and exist within a shared pressurized space; the simulator cannot generate different pressurized environments for the two lungs, and thus, it cannot replicate conditions such as a unilateral pneumothorax. Our simulator successfully replicates the physiologic tidal volume; however, it is not capable of generating the extremes of breath volumes. This is due to both the rigid ribcage and the simplified mechanism of diaphragm motion. Our simulator assumes rigid geometries of the rib cage; although the diaphragm is responsible for the majority of motion, the lack of accessory muscle and rib cage motion does not replicate the expiratory effort of rib cage collapse and limits the maximum volume inspired. Furthermore, the non-active diaphragm membrane of the diaphragm is not capable of generating the extremes of breath volumes. Because the diaphragm is pulled down via McKibben actuators in this simulator, it also does not accurately capture diaphragm contractile motion. Our flow waveforms show oscillating artifacts especially in the lower pressure ranges resulting from the electropneumatic regulator unit. This can be attributed to the pressure difference between the regulator input and output, as well as the compliance and resistance of the pneumatic circuit connected to the output. In our current applications, the volume of the output circuit is much greater than that of the pressure regulator, forcing the regulator to draw more pressure from the pressure source, with high wall pressures of 414 kPa. Our exact pressure output to the PAMs is dependent on the internal proprietary control scheme of the regulator unit that has an inherent rise time of 100 ms, which is slow enough to allow for overshoot and ensuing counteraction. This behavior has a minor impact on our overall flow, simulated lung respiratory volumes, and pleural and abdominal pressures. Approaches to mitigate these characteristics include (i) introducing a greater capacitive component to the output circuit, (ii) minimizing necessary volume of the output circuit, (iii) using a pressure regulator with a smaller range of output pressures to increase the set point pressure resolution, and (iv) using a pressure source that is closer to the maximum required output pressure.

Future work for this simulator includes expanding the biomimetic capabilities of the simulator. To increase fidelity of diaphragm action, we aim to develop a biomimetic active diaphragm. Using a system that physically replicates the thoracic and abdominal cavities, we can create interchangeable diaphragm inserts and corresponding soft robotic actuators that replicate physiologic and pathologic breathing pressures. The diaphragm mechanics of different pathologies can be quite varied because diaphragm dysfunction arises from a range of etiologies and are accompanied by changes in mechanical properties of the tissue such as stiffening or thickening. This would have the ability to model different types of pathology through varying material properties and actuation strategies. To investigate the relationships of the dynamic negative pressures of the thoracic cavity with the cardiovascular system, the system can be integrated with an active biohybrid heart and a cardiovascular flow loop. This would realize a full cardiopulmonary simulator of the thoracic cavity, allowing for both a key educational opportunity and also a new platform for medical device testing. These are *in vitro* platforms that have never been explored.

Ultimately, this simulator can be educationally independent or complementary to existing mannequin simulators. In low resource settings, our simulator provides a much more accessible yet robust simulator alternative to the expensive mannequin simulators. In high resource educational environments, our simulator will provide the preparatory mental models that allow students to gain the most out of the clinically realistic scenarios that can be generated via mannequin-based simulators. The accurate replication of the mechanics of the respiratory system will also act as a key *in vitro* testbed for mechanical medical device testing and rapid prototyping. This respiratory simulator exhibits the advantages conferred by focusing on replicating physiological biomechanics instead of developing a simulator geared toward a specific application.

## V. METHODS

We constructed the two main cavities simulating the thoracic and abdominal space from acrylic (polymethylmethacrylate) plates of $\frac{1}{4}$ in. thickness, which were fixed with screws and sealed with chemical adhesive (Clear Weld by JB Weld). The simulated abdomen included a window composed of a silicone sheet that serves as the compliance element (DragonSkin FxPro, Smooth-on, Inc.). To vary the compliance, we covered the silicone window with a layer of non-extensible polyester and attached it with Velcro around the perimeter; it can be rolled back to various degrees to expose the silicone window and, therefore, increase the compliance as desired.

We formed the shape of the rib cage that borders the modeled diaphragm (the zone of apposition) using a thermal vacuum forming machine (Formech450DT, Formech, Inc.). For this purpose, we segmented computer tomographic images of a ribcage using image processing software (Mimics, Materialise NV). The same data were used to cast a model of the corresponding diaphragm in a composite material of silicone and nylon mesh (DragonSkin20, Smooth-Sil, Inc.). We selected these silicone elastomers based on the fact that they were readily available in a range of mechanical properties to match our design requirements and our previous experience with using silicone to mimic dynamic features of dynamic tissue.^22–24^ We 3D printed the upper region of the rib cage that contains the lungs, with geometry derived from physiologic data (Dremel 3D45). To generate the contractile force that displaces the diaphragm down toward the abdominal space, we used McKibben pneumatic artificial muscles. Six of these actuators are equally distributed, sutured, and sealed through the silicone and nylon diaphragm (Silpoxy, Smooth-Sil, Inc.). On the opposite side, the actuators are screwed to the bottom face of the abdominal cavity. We fabricated the McKibben actuators as previously reported^22–24^ and added a latex outer sleeve to facilitate emptying of the pneumatic elements after contraction.

Due to their similarity in size, anatomy,^25^ and lung tissue resistance and elastance,^26^ we used swine lungs. Ethics approval for using *ex vivo* swine tissue was obtained from the Committee on Animal Care at the Massachusetts Institute of Technology (Protocol 0118–016). We used swine lungs fixed in a solution based on propylene glycol to preserve tissue elasticity and used cyanoacrylate-based adhesive to seal any residual tissue defects (AnatomyWarehouse). To decrease lung compliance, we tightly enclosed one side of the lungs in a silicone sleeve as well as a thermoplastic elastomer sleeve (C2 and C3 in Fig. 5). To alter compliance of the simulated pleural space, we filled the cavity with incompressible water containers displacing the compressible air surrounding the rib cage. We narrowed the airflow channel using a ball valve to increase airway resistance. We created a closed, open, and tension pneumothorax by elevating the initial negative pleural pressure in the chest cavity, opening the pleural space to air, or increasing the initial pleural pressure above atmospheric values.

For all ventilator related experiments, we used the corresponding tube circuit of the ventilator system to connect with our respiratory simulator (Hamilton G5, Hamilton Medical). We used a surgical blade to create a 5 mm lung puncture, which we sealed using a tissue sealing device as previously described.^27^ We measured flow and pressure before and after the sealing procedure. The tidal volume at the baseline was measured. Once the lungs were punctured, the tidal volume was recorded for subsequent breaths and expressed as a percentage of the initial tidal volume. Similarly, when the puncture was sealed, subsequent breaths were expressed as a percentage of baseline and overlaid in a bar chart as shown in Fig. 9(d).

All measurements were obtained using a mass flow meter and pressure sensors connected to signal conditioning and a custom software interface (SFM3000 by Sensirion AG, ArgoTrans model2 by Argon Medical Devices, NI9237 and Labview software by National Instruments, Inc.).

We controlled actuation patterns of the diaphragm with a customized pneumatic control system shown in Fig. S1. We delivered pressurized air by inputting a pressure waveform to an electropneumatic pressure regulator. We used a laptop with a custom user interface (designed in “Processing” programming language) that communicated input values to the developer board (Arduino Mega). The microcontroller on the developer board inputs the desired pressure waveform to the electropneumatic pressure regulator (ITV1030, SMC, Inc.), which was connected to the wall compressed air supply. We simultaneously used the microcontroller to open and close a solenoid valve (NVKF333, SMC, Inc.) using a 12 V MOSFET that allowed the pressure regulator to provide the desired pressure wave form to actuate the pneumatic artificial muscles and move the diaphragm in our model.

## SUPPLEMENTARY MATERIAL

See the supplementary material for a schematic of the customized pneumatic control system (Fig. S1).

## AUTHORS' CONTRIBUTIONS

M.A.H. and L.H. contributed equally to this work.
